# Proteomics reveals coordinated stress adaptation by a MazF toxin to conserve carbon, sustain central metabolism, and preserve PDIM biosynthesis in *Mycobacterium tuberculosis*

**DOI:** 10.1128/msystems.01814-25

**Published:** 2026-03-23

**Authors:** Bruno L. Abbadi, Valdir C. Barth, Safreen Sain, Julia Puffal, Jumei Zeng, Parth K. Patel, Robert N. Husson, Nancy A. Woychik

**Affiliations:** 1 Department of Biochemistry and Molecular Biology, Rutgers-Robert Wood Johnson Medical School 332317, Piscataway, New Jersey, USA; 2 Division of Infectious Diseases, Department of Pediatrics, Boston Children’s Hospital and Harvard Medical School 546608, Boston, Massachusetts, USA; University of Southampton, Southampton, United Kingdom

**Keywords:** proteomics, lipid, tuberculosis

## Abstract

In response to host-generated stresses, *Mycobacterium tuberculosis* (Mtb) reprograms its physiology in myriad ways to establish and maintain an infection, yet the signals that underlie this transformation are not well defined. The abundant toxin-antitoxin (TA) systems harbored in the Mtb genome, including 11 in the *mazEF* family, are thought to act as stress sensors, yet their roles are largely unknown. Although TA systems from other bacteria are generally thought to impart reversible growth arrest in response to stress, the exquisite specificity of Mtb tRNase toxins instead portends a more nuanced role. Here, we used a proteomics approach to track *de novo* protein synthesis to uncover molecular events initiated by the Mtb MazF-mt9 toxin (MazF7, Rv2063A). First, we documented striking enrichment of enzymes and transporters derived from the contiguous 36-gene region for phthiocerol dimycocerosate (PDIM) synthesis without an accompanying increase in PDIM lipid production. This paradox was reconciled by concomitant downregulation of proteins comprising the Mce1 transporter (imports host fatty acids), cholesterol breakdown, and β-oxidation enzymes (limiting the PDIM precursor methylmalonyl-CoA). Thus, increased catalytic efficiency of the PDIM pathway appears to offset substrate starvation to ensure adequate production of PDIMs essential for Mtb early immune escape and virulence. Finally, isocitrate lyase 1 levels also increased, which in this context are expected to primarily catalyze the glyoxylate shunt to sustain central carbon metabolism while minimizing carbon loss. These exacting proteomic signatures are paralleled within the bedaquiline-treated Mtb transcriptome, highlighting a critical role for MazF-mt9 in orchestrating Mtb stress survival.

**IMPORTANCE:**

The bacterial pathogen that causes tuberculosis, *Mycobacterium tuberculosis* (Mtb), must survive a gauntlet of immune assaults to establish an infection. Here, we determined that in response to host-imposed stresses, this pathogen enlists the action of a tRNase, the MazF-mt9 toxin, to reprogram the translatome and orchestrate metabolic remodeling to ensure adequate production of specialized phthiocerol dimycocerosate (PDIM) lipids on the cell surface, which contribute to early immune evasion. This toxin also upregulates isocitrate lyase 1 as a complementary survival-oriented adaptation that conserves carbon and sustains central metabolism for essential cellular functions. Thus, this toxin-mediated cooperative reprogramming toward preservation of PDIMs and central metabolism under lipid precursor-limiting conditions likely enables Mtb to successfully infect and survive in the host lung. Overall, the MazF-mt9-mediated protein expression signatures align with the transcriptome signatures of Mtb cells during bedaquiline treatment, suggesting a precise and essential role for this toxin in Mtb stress survival.

## INTRODUCTION

Over the millennia, *Mycobacterium tuberculosis* (Mtb) has adapted to evade killing by the multitude of assaults emanating from the host immune system or by treatment with antituberculars. One of many adaptations is the acquisition of 67 type II toxin-antitoxin (TA) systems in the Mtb genome (1, 2). Type II TA systems are operons comprising adjacent genes encoding two small (~10 kDa) proteins, a toxin and its cognate antitoxin that inhibits toxin activity through formation of a stable TA protein-protein complex. Stress conditions are thought to lead to lower levels of the antitoxin bound to the toxin and thus, a preponderance of free toxin, which then acts on its target inside bacterial cells to modify Mtb physiology to enhance cell survival (3). Emerging studies suggest a structural change in the TA complex underlies release of the antitoxin and toxin (reviewed in reference 4). This is consistent with data from the Husson laboratory demonstrating individual phosphorylation of two Mtb VapB antitoxins prevents association with their cognate toxins (5).

The Mtb genome contains 11 MazEF (MazE antitoxin, MazF toxin) TA modules (2, 6), whereas *Mycobacterium smegmatis*, *Escherichia coli,* and most other free-living bacteria contain just one (1, 2, 7). It is not understood why Mtb acquired so many MazEF modules. Biochemically, all bacterial MazF toxins are single-strand, sequence-specific endoribonucleases. We have previously determined the enzymatic activity of four Mtb MazF toxins. MazF-mt3, MazF-mt6, and MazF-mt11 inactivate rRNA through cleavage at distinct five-nucleotide recognition sequences within single discrete functionally essential domains (6, 8–10). By contrast, MazF-mt9 is a tRNase that inactivates one tRNA, tRNA^LysUUU^, by severing it in half at its anticodon sequence (11–13). MazF-mt9 thereby causes transcriptome-wide ribosome stalling at cognate AAA lysine codons because tRNA^LysUUU^ is depleted. Consequently, transcripts harboring these stalled ribosomes are cleaved and removed from the transcriptome, as shown by introducing MazF-mt9 into the model mycobacterium *M. smegmatis* as proof of principle (11). The impact of MazF-mt9—or any MazF toxin—on Mtb physiology has yet to be investigated.

Here, we enlisted a quantitative proteomics approach that tracks *de novo* protein synthesis upon MazF-mt9 expression to illuminate how this toxin precisely reprograms Mtb cells. We documented up- and downregulation of *de novo* synthesis of proteins belonging to only a handful of interrelated pathways that suggest toxin-mediated preservation of central metabolism and phthiocerol dimycocerosate (PDIM) levels when precursor lipids are scarce. Our constellation of proteomic findings aligns with documented hallmarks of Mtb subjected to certain host stresses or antibiotics, placing MazF-mt9 as a key mediator of the metabolic changes essential for Mtb survival in response to a variety of stresses it must endure to establish and maintain an infection in the human host.

## RESULTS

### MazF-mt9 reprograms the Mtb translatome

We had previously shown that MazF-mt9 does not inhibit protein synthesis (11). Instead, depletion of the sole tRNA^LysUUU^ target of MazF-mt9 leads to genome-wide stalling at cognate Lys AAA codons in Mtb and *M. smegmatis*. Transcripts harboring these stalled ribosomes are subsequently cleaved and degraded in *M. smegmatis*, resulting in proteome remodeling (11). In this work, we sought to understand how MazF-mt9 affects the Mtb translatome and determine if these alterations reveal clues to how this toxin alters cell physiology and/or influences the virulence of this pathogen. To do this, we used a proteomic approach that enables us to track and quantify newly synthesized proteins after toxin expression using mass spectrometry.

Since the natural triggers of toxin expression are not known, we used the TetR–*tet*O operator-based, anhydrotetracycline (ATc)-inducible pMC1s integrating plasmid designed by Ehrt et al. for highly specific, low-level inducible expression in Mtb (14). The TetR–*tet*O operator is one of the most efficient inducible systems for controlling gene expression due to the exceptionally specific, high-affinity binding of tetracycline or nontoxic analogs such as ATc to TetR (15). Consequently, the ATc inducer cannot randomly bind to other proteins to cause nonspecific effects because the affinity of ATc for TetR is higher than that of any other interaction in a cell (16). Ehrt et al. also engineered pMC1s for low expression by placing the Tet repressor under the control of a strong promoter, thus ensuring that there is more TetR expressed than can be relieved by the addition of ATc (14). Therefore, there is no unbound ATc available to randomly bind to other Mtb proteins and cause collateral damage. In fact, there are no native Mtb target genes that resemble a Tn10 *tet*O that could be inadvertently repressed by TetR.

**Fig 1:**
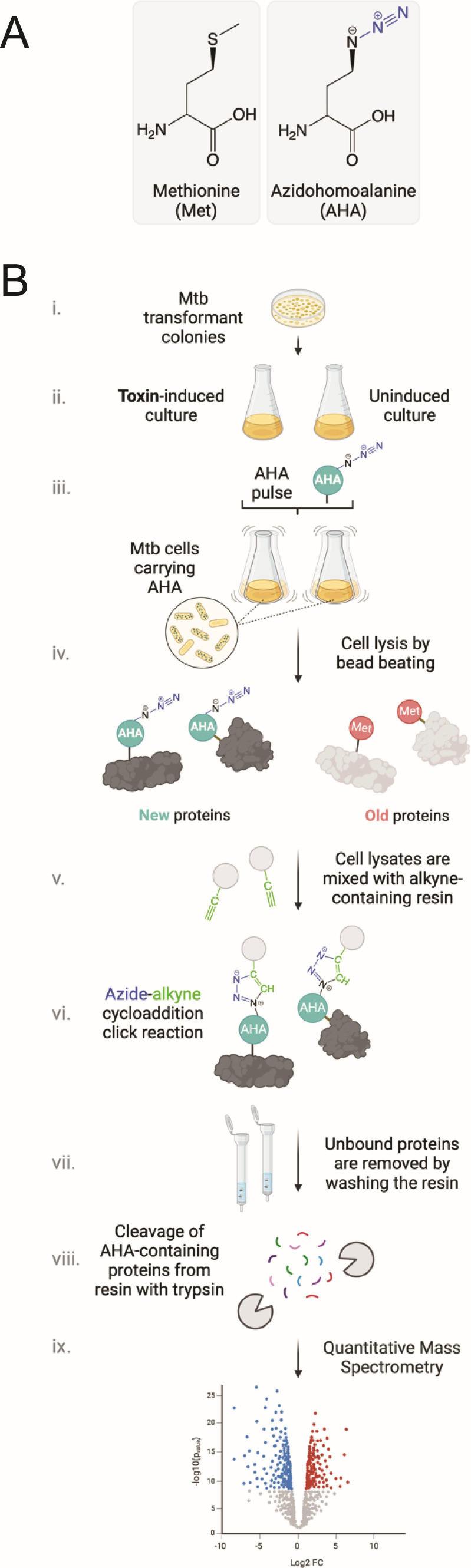
Labeling and capture of newly synthesized Mtb proteins for mass spectrometry using BONCAT. (**A**) Molecular structure of AHA, a methionine (Met) analog carrying an azido moiety. (**B**) Capturing newly synthesized, AHA-containing proteins by azide-alkyne cycloaddition click chemistry. AHA is added to actively growing cultures for a defined period and is incorporated into nascent proteins. A resin containing an alkyne group is then used to specifically capture AHA-containing proteins from cell lysates. Bound proteins are washed several times to remove proteins that do not contain AHA. The remaining proteins are cleaved by trypsin, followed by elution of tryptic peptides, which are then analyzed by quantitative mass spectrometry. The effect of the toxin on the translatome is determined by genome-wide comparison of newly synthesized proteins derived from uninduced versus toxin-induced cultures. Created with BioRender.com.

**Fig 2:**
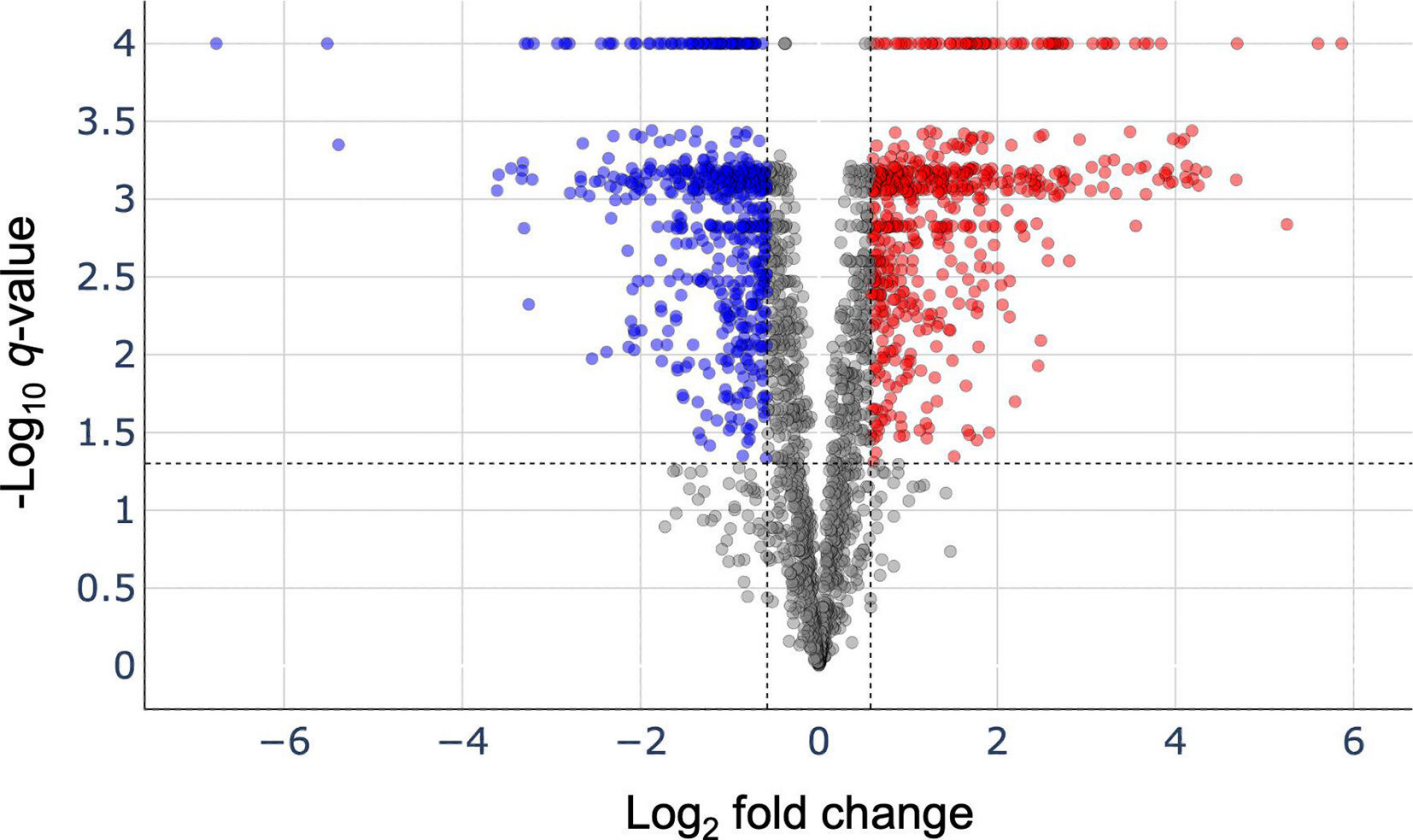
MazF-mt9 reprograms the Mtb translatome. Volcano plot illustrating up- and downregulation (log_2_ of fold changes) of newly synthesized AHA-labeled proteins following MazF-mt9 expression relative to the uninduced control. Of the 2,332 proteins detected by quantitative mass spectrometry, 1,132 were differentially expressed (575 upregulated, red dots; 557 downregulated, blue dots; ±1.5-fold) and statistically significant (*q*-value ≤0.05). Horizontal and vertical dotted lines indicate significance cut-offs of *q*-value = 0.05 and log_2_ fold change ±0.58 (corresponding to ±1.5-fold), respectively.

In agreement with the engineered properties of pMC1s, we previously reported modest 4- to 6-fold toxin expression following ATc induction relative to the uninduced control (11, 17–19). To track newly synthesized proteins, we used a nonradioactive *in vivo* metabolic labeling method called bioorthogonal noncanonical amino acid tagging (BONCAT, Fig. 1 [20, 21]). BONCAT incorporates a noncanonical form of methionine that contains an azido group, azidohomoalanine (AHA), into nascent proteins (Fig. 1A) to enable capture of newly synthesized proteins in control versus toxin-expressing Mtb cells (Fig. 1B, i to iv). Consistent with Ehrt et al. and our publications using pMC1s for expression of VapC Mtb toxins, the level of free MazF (not bound by MazE antitoxin) in these BONCAT samples is 2.7-fold higher than the uninduced control (Table S1). Newly synthesized, AHA-containing proteins were covalently captured via Cu(I)-catalyzed azide–alkyne click chemistry onto an alkyne resin (Fig. 1B, v and vi). The resin and bound proteins were placed in a column, washed, and the captured proteins released from the resin by trypsin (Fig. 1B, vii and viii). Finally, the isolated tryptic peptides were identified and quantified by mass spectrometry (Fig. 1B, ix). Among the 2,332 newly synthesized proteins detected, 1,132 were statistically significant and differentially expressed—575 upregulated (red dots, Fig. 2) and 557 downregulated (blue dots, Fig. 2)—upon MazF-mt9 expression for 9 days (Table S1).

### MazF-mt9 stimulates synthesis of proteins belonging to two functional themes

**Fig 3:**
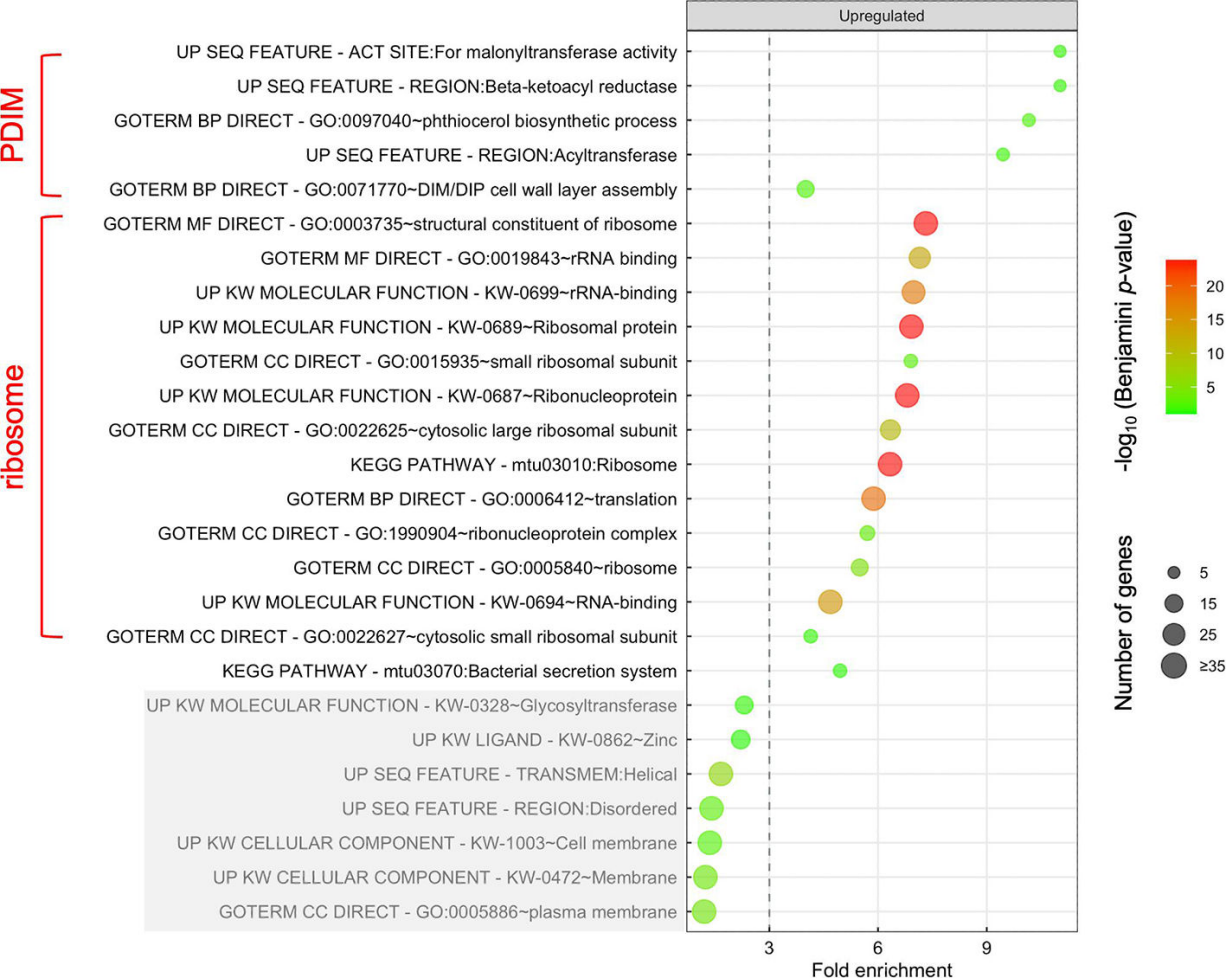
MazF-mt9 mediates an increase in synthesis of PDIM pathway enzymes and ribosomal proteins. DAVID Functional Analysis Tool terms (22, 23) associated with proteins significantly upregulated (*q*-value ≤0.05; log_2_ FC ≥ 1) following MazF-mt9 expression. Circle color corresponds to its −log_10_ Benjamini *p*-value, and circle diameter reflects the relative number of proteins in the corresponding category. Annotation categories that contain overlapping or identical proteins were grouped into two functional themes (red brackets). The bottom seven categories (shaded in gray) do not meet our significance cutoff (≥3.0) for fold enrichment.

To gain insight into MazF-mt9 function in Mtb, we used the Database for Annotation, Visualization and Integrated Discovery (DAVID) functional annotation tool to determine if there were protein families in our mass spectrometry data set that were enriched or depleted upon MazF-mt9 expression (22, 23). Among the 575 upregulated proteins, DAVID analysis identified subsets of proteins that sorted into 19 annotation categories (Fig. 3; Table S2, Upregulated tab). However, in many instances, we found that proteins within different annotation categories were either identical or overlapped (e.g., “ribosomal protein” versus “translation”). Therefore, these protein annotation categories were distilled into two predominant functional themes representing discrete pathways: PDIM biosynthesis/transport and ribosome biosynthesis (Fig. 3).

### Enhanced ribosomal protein production offsets toxin-mediated ribosome stalling

**Fig 4:**
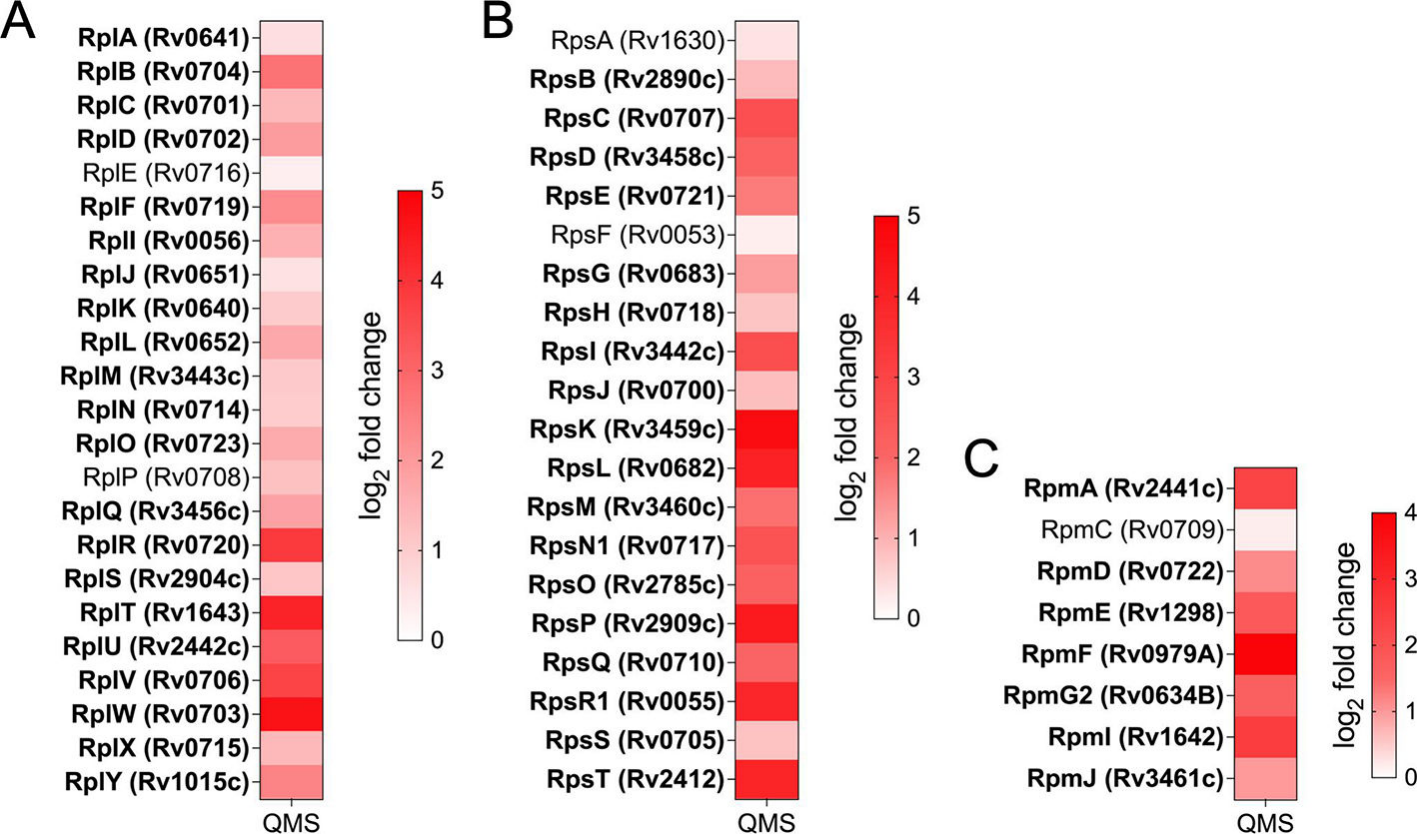
MazF-mt9 expression leads to enhanced ribosomal protein synthesis. Heat maps of the 51 ribosomal proteins (ribosomal protein name and Rv number are shown for each) detected by BONCAT; 6 of the 57 Mtb ribosomal proteins were not detected. Ribosomal proteins in the three panels are either (**A**) “Rpl” proteins in the large 50S ribosomal subunit, (**B**) “Rps” in the small 30S ribosomal subunit, or (**C**) “Rpm” proteins, also in the large 50S ribosomal subunit. Log_2_ fold changes were determined by comparing uninduced vs toxin-induced cultures. The 46 ribosomal proteins that increased ≥1.5-fold (log_2_ fold change ≥0.58) and were statistically significant (*q*-value ≤0.05) are shown in bold.

We detected 51 of the 57 Mtb ribosomal proteins by quantitative mass spectrometry; 46 of the 51 detected (90%) were upregulated and statistically significant upon MazF-mt9 induction (Fig. 4). Although the increase in ribosome synthesis seems counterintuitive since MazF-mt9 is causing cell growth arrest, this is happening in a cell whose free ribosome population is challenged by the action of this toxin. As we previously documented, MazF-mt9 has a single direct target—it is an isoacceptor-specific tRNase that only cleaves and depletes one tRNA, tRNA^LysUUU^. This has genome-wide consequences for ribosome availability. Depletion of tRNA^LysUUU^ leads to transcriptome-wide ribosome stalling at AAA codons (11). Therefore, increased synthesis of ribosomal proteins appears to be triggered to counteract the resulting shortage of free ribosomes and keep their levels above the critical threshold for cell survival. Although ribosomes are likely recycled upon stalling—we detect mRNA cleavage at the 5′ side of these stalled ribosomes (11)—the resulting flux in available ribosomes constitutes ribosome stress. In response, it is logical that Mtb cells would seek to compensate by ratcheting up synthesis of ribosomal subunits to maintain normal ribosome levels. To assess the toxin’s effect on overall ribosomal protein abundance, we performed steady-state quantitative mass spectrometry instead of BONCAT from control cells and those expressing MazF-mt9 and found no statistically significant change in the abundance of any ribosomal protein detected (data not shown). These steady-state data support our hypothesis that increased new ribosomal protein synthesis in our BONCAT data set represents replenishment of depleted ribosomes and not overall enhancement of ribosome levels.

### MazF-mt9 selectively enhances synthesis of enzymes and transporters for PDIM synthesis

The waxy, thick, protective Mtb cell envelope has several layers, many of which have been shown to be essential for viability or for virulence in animal models (reviewed in references 24–26). The cytoplasmic membrane forms the innermost layer, followed by the peptidoglycan layer, the arabinogalactan layer, and the mycolic acid layer. The next layer is composed of several complex lipids: sulfolipid-1 (SL-1), diacyl trehalose (DAT), and polyacyl trehalose (PAT), trehalose mono-mycolate (TMM), trehalose di-mycolate (TDM), phosphatidylinositol mannoside (PIM), lipomannan (LM), lipoarabinomannan (LAM), mannosylated lipoarabinomannan (ManLAM), phenolic glycolipids (PGLs) in strains with an intact *pks15/1* gene, and PDIMs. Finally, the outermost layer of the Mtb cell envelope is the capsule composed mainly of a glycogen-like α-glucan along with arabinomannan, mannan, other lipids, and proteins.

**Fig 5:**
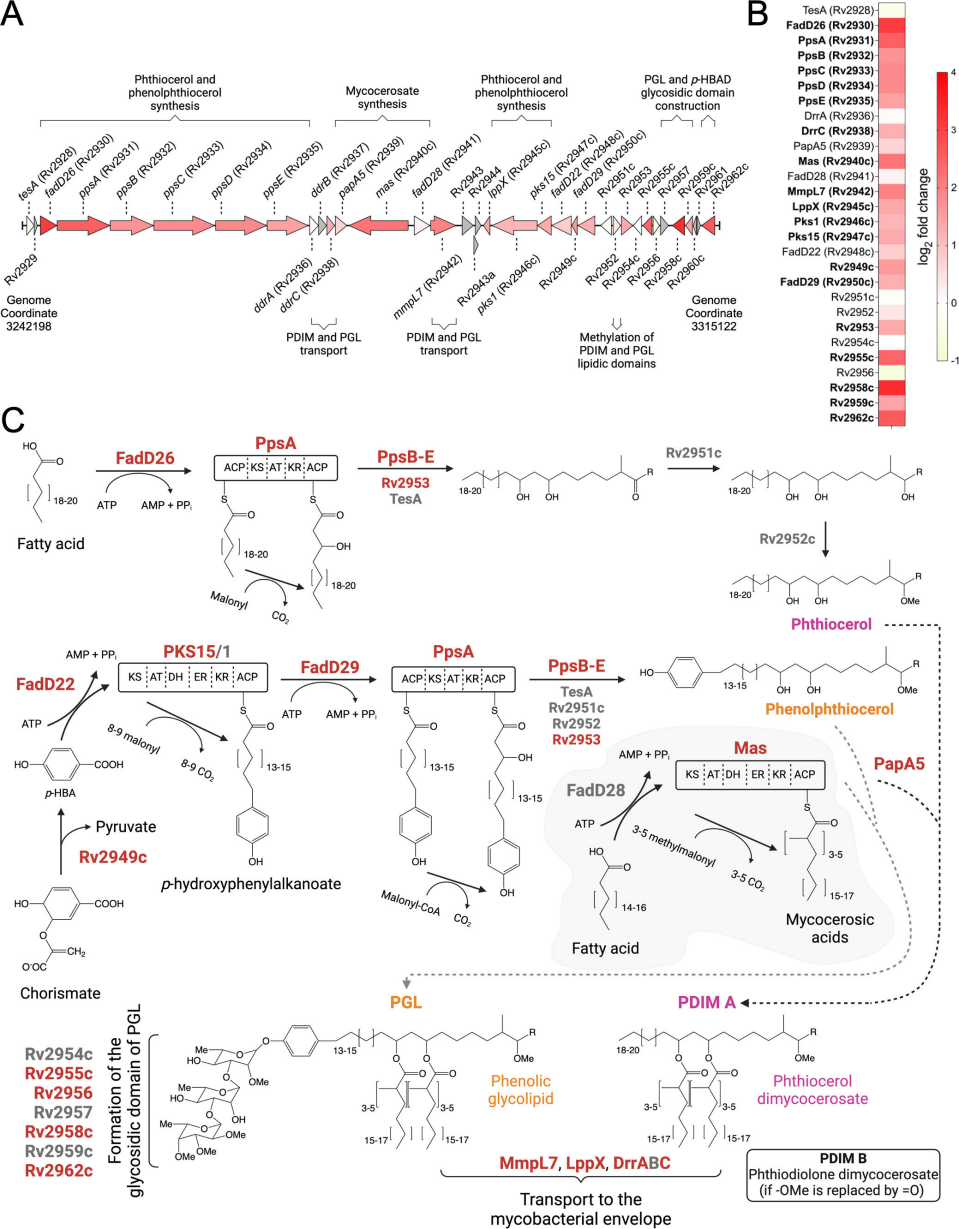
MazF-mt9 selectively enhances synthesis of enzymes and transporters for PDIM synthesis. (**A**) Mtb PDIM and PGL gene cluster. Upregulated proteins (shades of red in the horizontal arrows) are colored to match the corresponding colors in the heatmap in panel B. Gray arrows represent proteins undetected by quantitative mass spectrometry. The genome coordinates indicating the 5′ and 3′ ends of the represented chromosomal segment (72,925 bp total) are shown. Genes depicted as arrows; sizes approximate and not to scale. (**B**) Heatmap showing the log_2_ fold change of AHA-labeled, newly synthesized proteins involved in PDIM and PGL biosynthesis upon MazF-mt9 expression. Proteins detected by quantitative mass spectrometry upregulated ≥2-fold (log_2_ fold change ≥ 1) with a *q-*value ≤0.05 were deemed statistically significant and are shown in bold. (**C**) PDIM and PGL biosynthesis pathway in Mtb (30). Upregulated statistically significant, newly synthesized proteins are indicated by red text. KS, ketoacylsynthase; AT, acyltransferase; DH, dehydratase; ER, enoylreductase. KR, ketoreductase; ACP, acyl-carrier protein. Created with ChemDraw 22.2.0 and BioRender.com.

Five functional annotation categories associated with PDIM synthesis and transport to the Mtb cell envelope were enriched after MazF-mt9 induction (top of Fig. 3; reviewed in references 27–29). Proteins in this pathway are derived from the chromosomal region spanning 36 contiguous genes, Rv2928 (*tesA*) through Rv2962c (Fig. 5A). This region includes six functional polyketide synthase genes in H37Rv, *ppsA–E* and *mas*, that catalyze the synthesis of these specialized lipids (Fig. 5A). The MmpL7, LppX, and DrrA–C transporters are responsible for proper localization of PDIM across the cytoplasmic membrane to the cell envelope (Fig. 5A). Even though many proteins encoded by genes in this region were very small and thus difficult to detect by mass spectrometry, we were able to detect 28 of the proteins encoded by these 36 genes, 19 of which were upregulated and statistically significant after MazF-mt9 expression (shades of pink to red, Fig. 5A and B). The roles of each of these 19 proteins in the PDIM/PGL pathway are indicated by red text in Fig. 5C.

Note that even though we documented substantially increased synthesis of enzymes that catalyze PGL synthesis from PDIMs (Fig. 5A and B), we do not expect PGL synthesis to occur because of a known mutation in H37Rv and related strains that precludes synthesis of PGL. This stems from the lack of an intact *pks15/1* gene (versus the separate *pks15* and *pks1* genes in our strain shown in Fig. 5A) that encodes a polyketide synthase essential for PGL synthesis from phenylphthiocerol. The nonfunctional *pks15* and *pks1* genes result from either a 1 or 7 bp deletion in the intact *pks15/1* gene from H37Rv and derivatives compared to *M. bovis* BCG, which can synthesize PGL (31). We confirmed the presence of a 1 bp deletion in our H37Rv-derived strain upon comparison to the uninterrupted *pks15/1* gene in *M. bovis* BCG.

The three-carbon intermediate methylmalonyl-CoA serves as a key extender unit for the biosynthesis of PDIMs (Fig. 5C) and trehalose ester lipids SL-1, DAT, and PAT in the cell envelope. Yet, the effect of MazF-mt9 on Mtb cell lipids appears specific to PDIM/PGL synthesis. We did not detect evidence of upregulation of enzymes catalyzing synthesis of SL-1, DAT, or PAT. We also did not uncover compelling evidence for increased synthesis of proteins that participate in the synthesis and/or localization of peptidoglycan, mycolic acids, and PIMs. Therefore, the striking increases in enzymes catalyzing PDIM synthesis along with membrane proteins mediating its transport suggest that MazF-mt9 expression specifically increases production of PDIMs.

### PDIM lipids are maintained at wild-type levels despite enrichment of PDIM biosynthesis enzymes and transporters

**Fig 6:**
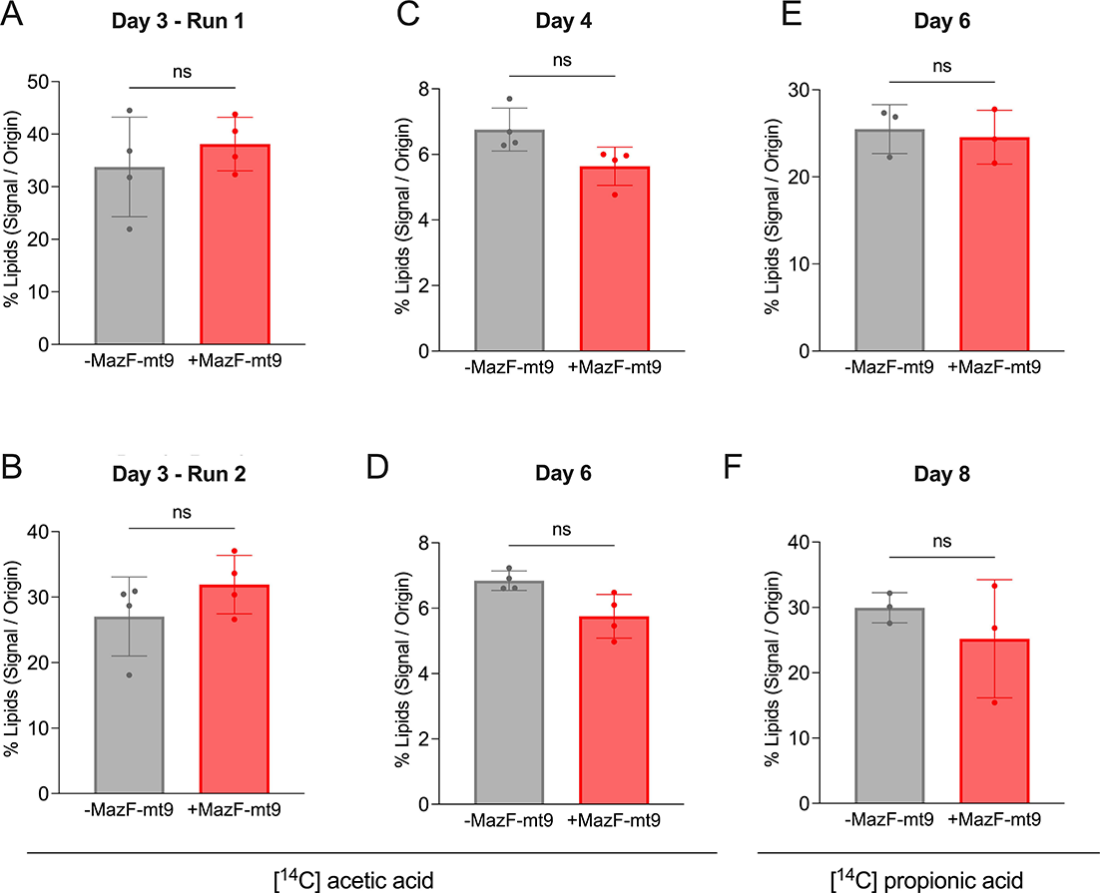
Wild-type levels of PDIMs are maintained despite upregulation of PDIM biosynthetic enzymes and transporters. Quantification of PDIM A (phthiocerol dimycocerosate) plus PDIM B (phthiodiolone dimycocerosate) from either [^14^C] acetic acid or [^14^C] sodium acetate-labeled Mtb cells after resolution by TLC. Histograms depict the relative total PDIM intensity upon MazF-mt9 expression (+MazF-mt9, red) compared to control levels (–MazF-mt9, gray). Error bars represent standard deviation; circles, biological replicates (*n* = 4 for panels **A–D** and *n* = 3 for panels **E, F**). “ns” indicates not significant when comparing the means of total PDIM abundance between uninduced and induced groups in a two-tailed, paired *t*-test comparison (**A, D–F**) or Wilcoxon matched-pairs signed-rank test (**B, C**).

To determine whether there was a corresponding increase in PDIM synthesis suggested by our proteomics data, we quantified PDIM levels in Mtb cells with or without MazF-mt9 induction. Cells were [^14^C] labeled, the lipids extracted, and the PDIMs resolved by thin-layer chromatography (TLC). We tried many different conditions: labeling with [^14^C] acetic acid or [^14^C] propionic acid as well as varying the length of toxin induction before [^14^C] labeling (Fig. 6A through F). Figure S1A through F provides a representative example of the PDIM quantification shown in Fig. 6, illustrating how toxin-induced cells with marginal growth and correspondingly poor [¹⁴C] uptake produce extremely weak PDIM TLC signals.

Across all conditions tested, we did not detect any statistically significant change in PDIM levels upon toxin expression. Mulholland et al. recently reported that *in vitro* Mtb culture selects for PDIM-negative mutations (32). However, among the panel of mc^2^ strains they tested, the mc^2^6206 strain used in this work exhibited normal PDIM levels (32). Consistent with Mulholland et al., we passaged our mc^2^6206 for several generations in media supplemented with 0.1 mM propionate and found no enhancement of PDIM recovery (data not shown). We were perplexed as to why PDIM levels did not increase despite the enrichment of PDIM pathway enzymes and transporters that we identified following MazF-mt9 induction. Therefore, we investigated the effects of MazF-mt9 on pathways upstream of PDIM biosynthesis to identify clues to resolve this paradox.

### Enhanced production of PDIM enzymes and transporters offsets reduced precursor availability to maintain PDIM levels

**Fig 7:**
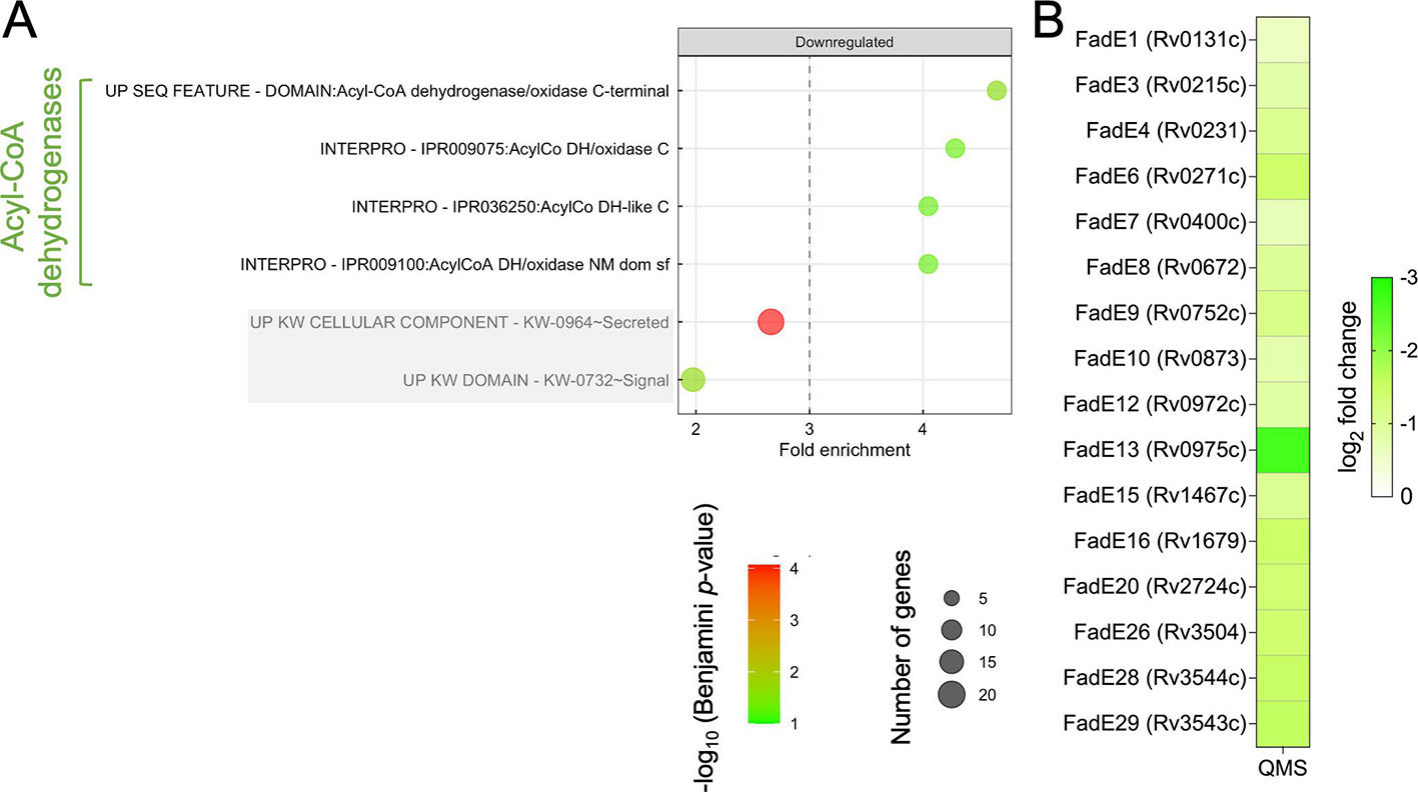
MazF-mt9 expression leads to reduced synthesis of several FadE proteins. (**A**) DAVID Functional Analysis Tool terms (22, 23) associated with proteins significantly downregulated (*q*-value ≤0.05; log_2_ FC ≤ −1) in the BONCAT data set following MazF-mt9 expression. Circle color corresponds to −log_10_ Benjamini *p*-value, and circle diameter reflects the relative number of proteins in the corresponding category. MazF-mt9 downregulates proteins belonging to four DAVID annotation categories. The bottom two categories (shaded in gray) do not meet our cutoff (≥3.0) for fold-enrichment significance. (**B**) Heat map of the 16 FadE proteins (probable acyl-CoA dehydrogenases) detected by quantitative mass spectrometry that were downregulated 1.5-fold or more (log_2_ fold change ≤ −0.58) and statistically significant (*q-*value ≤0.05).

DAVID analysis uncovered a single family of proteins, acyl-CoA dehydrogenases (ACADs), whose synthesis decreased upon MazF-mt9 expression. This family was represented in four annotation categories comprising identical subsets of FadE ACADs (Fig. 7A; Table S2, Downregulated tab). There are 35 Mtb proteins annotated as ACADs (33, 34). Our BONCAT data set contained 16 FadE ACADs with statistically significant false discovery rates (*q-*value ≤0.05), and all 16 were downregulated 1.5-fold or more (log_2_ fold change ≤ −0.58; Fig. 7B). The functions of the 35 Mtb FadE ACADs as they relate to Mtb physiology are not well studied, as is the case for prokaryotic ACADs in general. Nearly all Mtb FadEs are annotated as “function unknown; involved in lipid degradation.” Therefore, most Mtb FadE ACADs are assumed to catalyze a step in the β-oxidation pathway to produce acetyl-CoA or propionyl-CoA.

**Fig 8:**
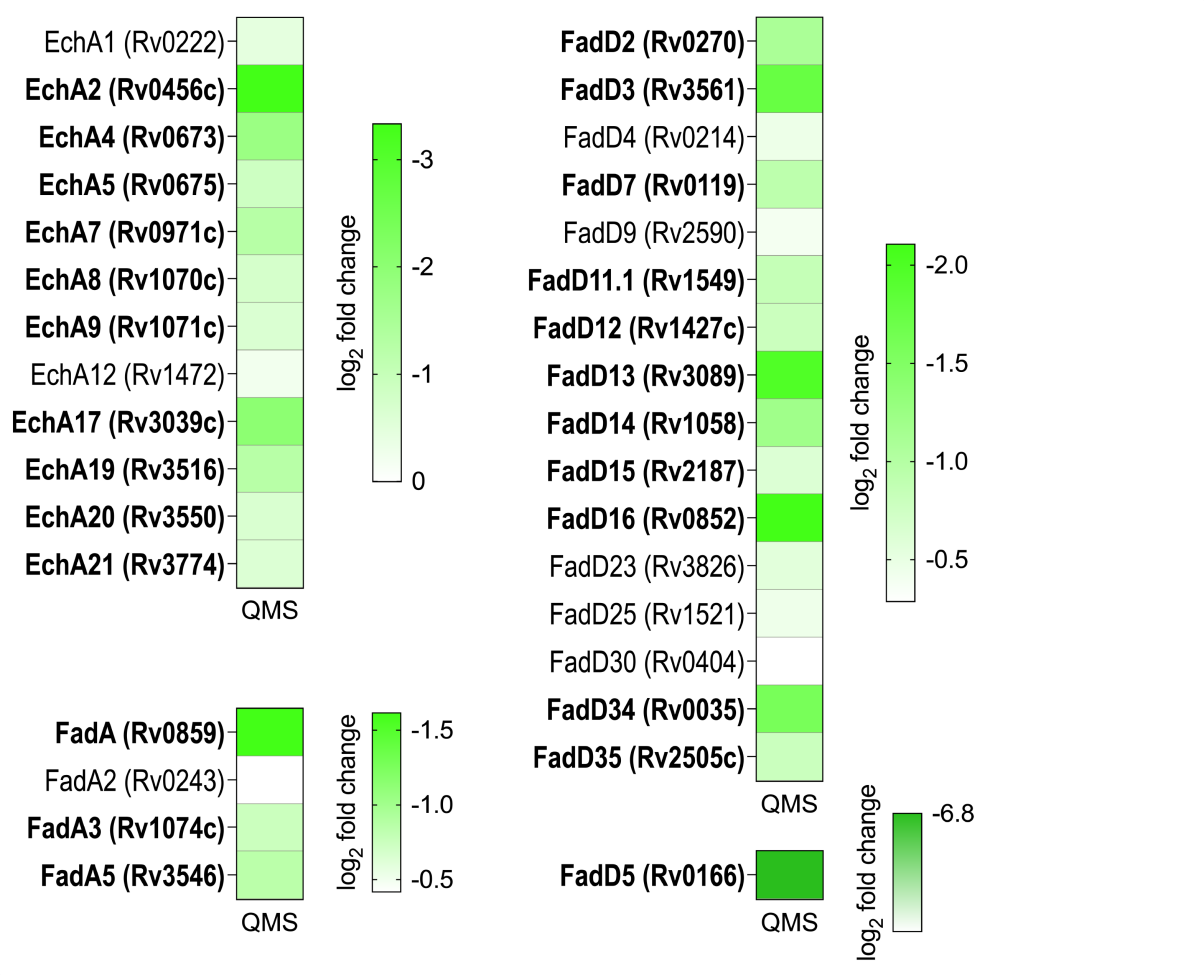
Synthesis of several other families of β-oxidation enzymes is also downregulated after toxin expression. Heat maps of enzymes detected in the BONCAT data set whose synthesis is downregulated and statistically significant (*q*-value ≤0.05) following MazF-mt9 expression. Bold text denotes those whose abundance decreased 1.5-fold or more (log_2_ fold change ≤ −0.58) and were statistically significant (*q*-value ≤0.05).

**Fig 9:**
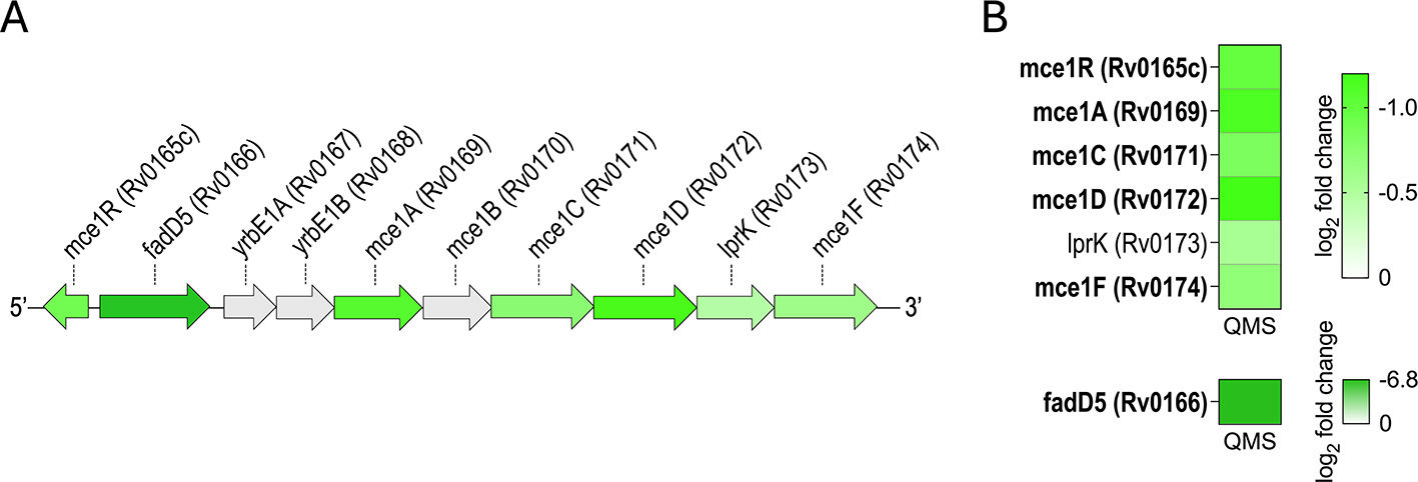
MazF-mt9 downregulates the synthesis of proteins comprising the Mce1 lipid transporter system. The *mce1* operon (**A**, approximately to scale) and heatmap (**B**) depicting all statistically significant (*q*-value ≤0.05) downregulated proteins in the BONCAT data set. Downregulated proteins in panel **A** (green horizontal arrows) are colored to match the corresponding colors in the heatmap in panel **B**. Bold text in panel **B** denotes statistically significant (*q*-value ≤0.05) proteins whose abundance decreased 1.5-fold or more (log_2_ fold change ≤ −0.58).

Since DAVID analysis identified a reduction in synthesis of FadE family members, we mined our BONCAT data set for other enzyme families that catalyze steps in β-oxidation. We found substantial decreases in synthesis of three additional enzyme families involved in β-oxidation: FadD, EchA, and FadA (Fig. 8A through C). We then checked proteins comprising the Mce1 ABC-like transporter system since β-oxidation breaks down fatty acids imported from the host. Although Mce1 can import both even- and odd-chain fatty acids, essentially all available fatty acids are even-chain in human hosts. β-Oxidation of these even-chain fatty acids is a major source of acetyl-CoA. The abundance of most proteins encoded by the Mce1 operon decreased after MazF-mt9 expression (Fig. 9A and B). Although Mce1R has been reported to function as a repressor of the *mce1* operon in early studies (35), it is divergently transcribed with the highly downregulated FadD5 protein (Fig. 9A). Since bidirectional promoters (which are typically coregulated) are conserved in all domains of life (36), Mce1R and FadD5 are expected to be coordinately regulated at the transcriptional level. In agreement, we detected a reduction in *de novo* protein synthesis of both Mce1R and FadD5 (Fig. 9A and B). However, while both FadD5 and Mce1R protein levels decline upon MazF-mt9 expression, it is unclear why FadD5 levels collapse precipitously (−6.8 log_2_ fold change; a 47-fold decrease) compared to Mce1R (−1 log_2_ fold change, a twofold reduction) (Fig. 9A and B).

**Fig 10:**
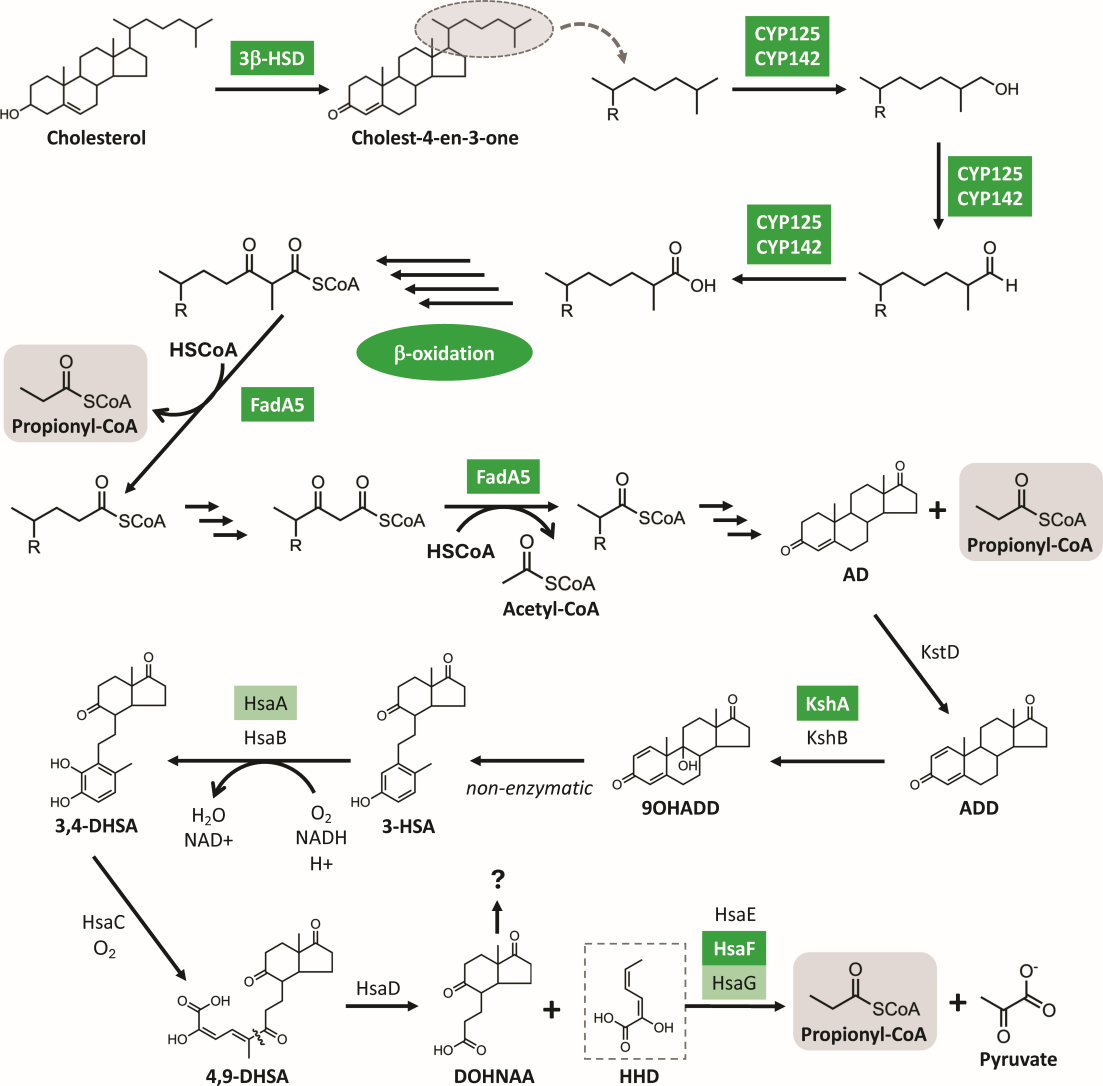
MazF-mt9 expression leads to the reduced synthesis of enzymes involved in cholesterol catabolism. Mtb cholesterol side-chain degradation pathway (37); steps following cholest-4-en-3-one only illustrate the fate of the acyl side chain (gray oval) with the remaining rings represented as “R.” Known enzymatic steps are shown; the triple arrows denote multiple undefined steps. The rings are once again shown on AD (4-androstenedione, middle right) after the second triple-arrow step. Dark green-boxed enzymes, down 1.5-fold or more (log_2_ fold change ≤ −0.58) and statistically significant (*q*-value ≤0.05); light green-boxed enzymes were downregulated to a lesser degree but also statistically significant (*q*-value ≤0.05). β-Oxidation represented here by four arrows, details included in Fig. 7 and 8. The three propionyl-CoAs generated in this pathway are boxed in gray for emphasis. The fate of DOHNAA is not known, while HHD (dotted box) generates propionyl-CoA and pyruvate. ADD, 1,4-androstenedione; 9OHADD, 9-hydroxy-1,4-androstene-3,17-dione; 3-HSA, 3-hydroxy-9,10-seconandrost-1,3,5(10)-triene-9,17-dione; 3,4-DHSA, 3,4-dihydroxy-9,10-seconandrost-1,3,5(10)-triene-9,17-dione; 4,9-DHSA, 4,9-dihydroxy-9,10-seconandrost-1,3,5(10)-triene-9,17-dione; DOHNAA, 9,17-dioxo-1,2,3,4,10,19-hexanorandrostan-5-oic acid; HHD, 2-hydroxy-hexa-2,4-dienoic acid. Created with ChemDraw 22.2.0.

We next interrogated cholesterol catabolism because cholesterol imported by another ABC-like transporter system, Mce4, is broken down into both odd- and even-chain fatty acids to produce the majority of Mtb propionyl-CoA and acetyl-CoA (with the former serving as the principal precursor for methylmalonyl-CoA in PDIM biosynthesis) (37). While we did not detect any significant changes in production of protein components of the Mce4 system in our BONCAT data set, there was significantly reduced synthesis of all three enzymes (3β-HSD, CYP125, and CYP142) involved in cholesterol side-chain degradation (Fig. 10). Another minor source of propionyl-CoA comes from degradation of branched-chain amino acids (BCAA), isoleucine, and leucine. There are five dedicated enzymes associated with propionyl-CoA synthesis from BCAA: IlvE, then BkdA, B, C, and ending with MmsA. *De novo* synthesis of IlvE (the gatekeeper of BCAA degradation) and MmsA (directly generates propionyl-CoA) is down more than 1.5-fold and statistically significant (*q-*values ≤0.001) upon MazF-mt9 expression. However, synthesis of BkdA and BkdC is significantly enhanced 1.6-fold and 3.3-fold, respectively (*q-*values ≤0.001; BkdB not statistically significant), making interpretation of the MazF-mt9 toxin on this pathway less clear.

**Fig 11:**
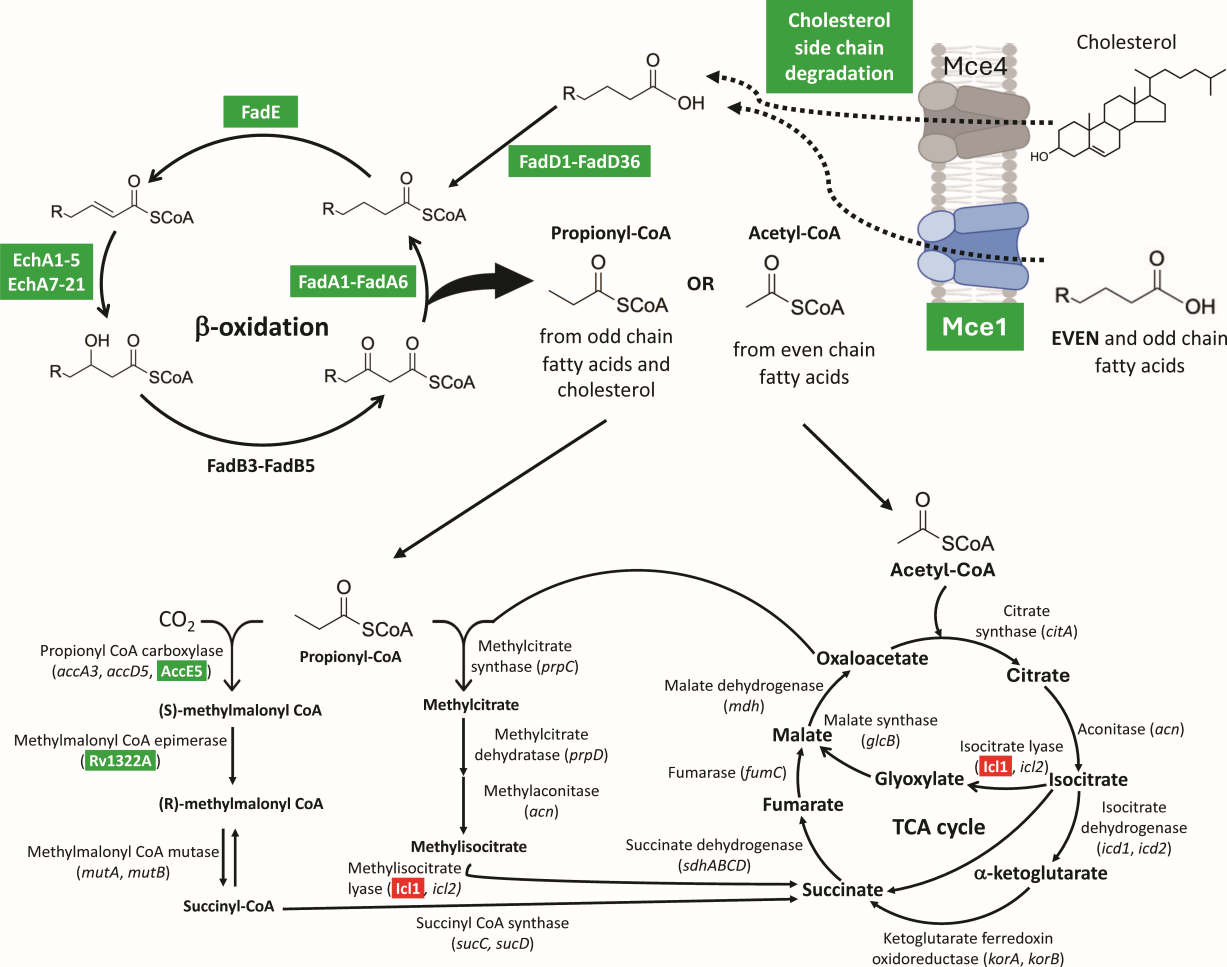
Model of MazF-mt9-mediated stress adaptation to conserve carbon and sustain central metabolism when PDIM precursors are limiting. Illustrated summary of the fates of imported lipids (cholesterol via Mce4, EVEN [capitalized to denote that it represents the majority of fatty acids in human hosts] and odd-chain fatty acids via Mce1). Text boxed with dark green represents enzyme families down 1.5-fold or more (log_2_ fold change ≤ −0.58) and statistically significant (*q*-value ≤0.05); β-oxidation enzymes detailed in Fig. 7 and 8. The sole upregulated enzyme, Icl1, with isocitrate lyase and methylisocitrate lyase activity is highlighted in red. Created with BioRender.com.

Collectively, our data support a model in which MazF-mt9 decreases the production of proteins across three interconnected pathways—Mce1-mediated fatty-acid transport, cholesterol catabolism, and β-oxidation—thereby limiting the availability of both propionyl-CoA and acetyl-CoA precursors required for PDIM biosynthesis (Fig. 11; green, downregulated proteins; red, upregulated). Propionyl-CoA provides the methyl-branched C3 precursor for PDIM, but acetyl-CoA supplies the even-carbon malonyl-CoA extender units used during polyketide synthesis of both the phthiocerol backbone (via PpsA–E) and the mycocerosic acids (via Mas). Thus, PDIM requires both acetyl-derived and propionyl-derived precursors for correct chain length, branching pattern, and overall lipid mass. Because the structural components of the Mce1 transporter, enzymes involved in cholesterol degradation, and multiple β-oxidation enzymes are reduced, the downstream generation of methylmalonyl-CoA from propionyl-CoA is also likely constrained. Consistent with this, levels of the propionyl-CoA carboxylase subunit AccE5 and the methylmalonyl-CoA mutase Rv1322A—enzymes that generate methylmalonyl-CoA—are significantly decreased (Fig. 11, bottom left). We, therefore, propose that the marked upregulation of PDIM biosynthetic enzymes and transporters reflects a compensatory response to precursor scarcity, consistent with the requirement for methylmalonyl-CoA in the first committed step of PDIM biosynthesis (Fig. 5C).

### MazF-mt9 drives robust synthesis of isocitrate lyase 1 (Icl1)

Alongside the increase in proteins in the PDIM pathway, we also detected a pronounced increase in *de novo* synthesis of the Icl1 enzyme—a 12.5-fold (3.65 log_2_ fold change) increase in protein abundance relative to the control—in response to MazF-mt9 expression. Icl1 has dual roles in Mtb: its canonical isocitrate lyase activity catalyzes the first step in the glyoxylate shunt branch off the TCA cycle (isocitrate to glyoxylate and succinate), and its methylisocitrate lyase activity converts methylisocitrate to succinate and pyruvate (Fig. 11, red highlighted text [38, 39]). Engaging the glyoxylate shunt bypasses the decarboxylation steps, preserving the two acetyl-CoA carbon units derived from lipid degradation for use in gluconeogenesis and biomass production. The alternate methylisocitrate lyase activity of Icl1 is in the last step in the methylcitrate cycle (Fig. 11, red highlighted text, lower middle); this cycle prevents toxic accumulation of propionyl-CoA and its derivatives. Of note, Icl1 has a functionally related isoform, Icl2, which was not detected in our BONCAT data set. Muñoz-Elías and McKinney noted that although both isoforms are upregulated in Mtb isolated from mouse lungs, only *icl1* is expressed *in vitro* (40). The dominance of Icl1 over Icl2 is supported by more recent studies indicating that Icl1 appears to serve as the major lyase isoform in *M. smegmatis* (41). This conclusion from *M. smegmatis* is extrapolated to the data derived from *in vitro* Mtb cultures because both *icl1* regulatory elements are conserved (41).

Early infection studies in mice using just a ∆*icl1* strain revealed that Icl1 is required for Mtb survival in the lungs during the persistent phase of infection but not in the acute phase (42). However, subsequent double *icl1/icl2* deletion studies indicated that both are required for Mtb growth, survival, and virulence in macrophages and mice (43). Given that Mtb primarily subsists on fatty acids during infection (40, 44), the essentiality of isocitrate lyase during Mtb growth on fatty acids was later defined in exquisite biochemical detail using chemogenetic and metabolomic approaches to track the consequences of loss of both isocitrate lyase activities (45). Finally, to link these metabolic changes to the Mtb response to drugs, the Rhee group demonstrated that Mtb exposure to first-line antibiotics with different targets (isoniazid, rifampicin, or streptomycin) all led to isocitrate lyase activation and antibiotic tolerance (46). Therefore, Icl1 occupies a pivotal metabolic node in Mtb pathogenesis. This enzyme is central to Mtb metabolic adaptation, and its substantial upregulation in response to MazF-mt9 indicates that toxin expression triggers metabolic remodeling that intersects directly with pathways essential for intracellular survival, persistence, virulence, and antibiotic tolerance.

### MazF-mt9 remodeling of the translatome parallels transcriptomic changes in response to specific stresses

**Fig 12:**
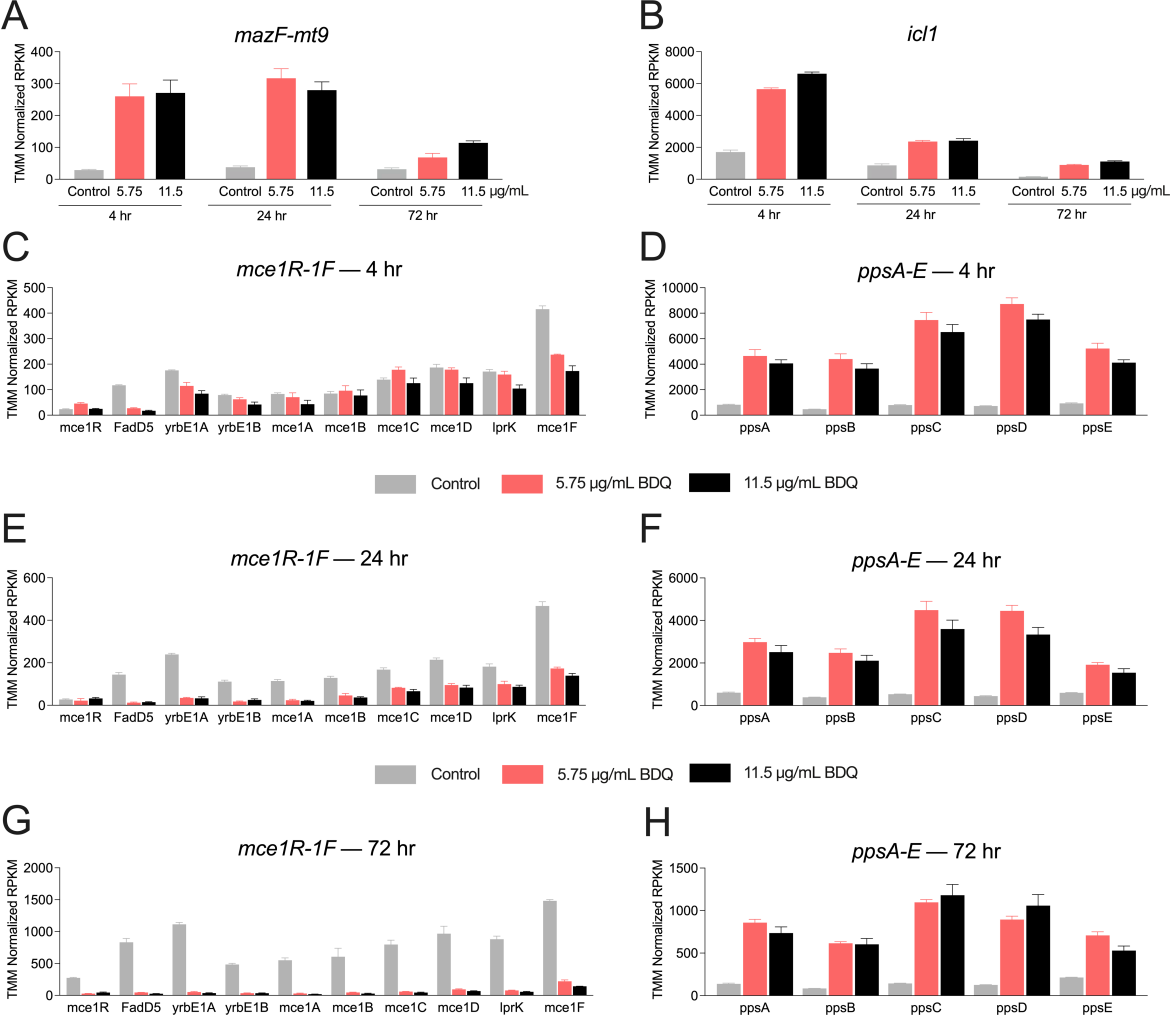
Toxin-mediated remodeling of the translatome parallels transcriptomic changes in response to bedaquiline. Bar graphs representing genes/gene families of interest derived from published Mtb H37Rv RNA-seq (47, 50, 51) from cells grown with or without bedaquiline (BDQ); control (gray), 5.75 µg/mL BDQ (salmon), 11.5 µg/mL BDQ (black) for the times (hr) shown. Error bars represent standard deviation of the mean. TMM, trimmed mean of M-values; RPKM, reads per kilobase per million mapped. (**A**) *mazF-mt9*; (**B**) *icl1*; (**C**, **E**, **G**) *mce1R-1F* (4, 24, 72 h, respectively); (**D**, **F**, **H**) *ppsA-E* (4, 24, 72 h, respectively).

To determine whether MazF-mt9–dependent remodeling of the Mtb translatome resembles cellular responses to known stresses, we compared our BONACAT proteomic data to published RNA-seq data sets derived from Mtb H37Rv subjected to diverse stress conditions (47, 48). Transcriptomic signatures from Mtb cells subjected to nitrosative stress (49), linezolid (50, 51), and bedaquiline (50, 51) were consistent with the changes in *de novo* protein synthesis outlined here in response to MazF-mt9. However, bedaquiline treatment most closely paralleled hallmarks of toxin expression using BONCAT. Transcripts for MazF-mt9, Icl1, and polyketide synthases PpsA–E in the PDIM pathway increased, while expression of genes encoding components of the Mce1 transporter system were downregulated (Fig. 12). This concordance suggests that MazF-mt9 triggers a stress-related transcriptional program that overlaps substantially with the response to bedaquiline, a drug known to disrupt energy metabolism. The similarity between these profiles highlights the extent to which MazF-mt9 reshapes central metabolic pathways and rewires Mtb physiology at the translational level.

## DISCUSSION

Our BONCAT analyses revealed coordinated remodeling of lipid metabolism upon MazF-mt9 induction. Proteins required for Mce1‐dependent fatty-acid uptake, cholesterol side-chain degradation, and downstream β-oxidation were broadly downregulated, thereby restricting both acetyl-CoA and propionyl-CoA flux into central metabolism and PDIM precursor pools. In contrast, synthesis of the key bifunctional enzyme Icl1 is strongly upregulated in response to MazF-mt9 induction, consistent with a compensatory response to altered lipid catabolism and a shift toward carbon-conserving or propionyl-detoxifying pathways. However, since propionyl-CoA levels are predicted to be limiting, Icl1 is expected to primarily act as an isocitrate lyase in the TCA glyoxylate shunt. MazF-mt9–expressing cells are in a carbon-saving, metabolism-preserving mode. Use of the glyoxylate shunt bypasses CO_2_-releasing TCA reactions and sustains central metabolism when acetyl-CoA and propionyl-CoA flux is reduced (Fig. 11), enabling cells to maintain an anaplerotic balance while multiple lipid catabolic pathways are repressed. Strikingly, despite reduced precursor-generating pathways, enzymes and transporters dedicated to PDIM biosynthesis are upregulated, suggesting toxin-dependent prioritization of PDIM assembly. This compensatory upregulation likely maintains PDIM output at wild-type levels even under conditions of limited precursor availability, highlighting PDIM as a metabolically protected virulence lipid during MazF-mt9–induced stress.

More broadly, the ability to quantify protein levels on a proteome scale can serve as a powerful predictive tool to unearth processes and pathways that are up- or downregulated by an effector molecule. Up- or downregulated proteins belonging to discrete cellular pathways or processes can accurately predict downstream events that align with the trend observed. For example, marked up- or downregulation of most/all enzymes in a pathway for synthesis of a key metabolite is expected to alter the synthesis of the metabolite accordingly. Likewise, enrichment or depletion of components of a protein complex/machine should increase or decrease its functional capacity. Even significant modulation of the levels of a single protein (such as a key regulatory protein) is expected to impact its downstream targets.

MazF-mt9 has a broad reach, and the effects on the PDIM pathway are likely indirect, given that the direct target of the MazF-mt9 toxin is extraordinarily specific—this toxin cleaves and inactivates a single tRNA^LysUUU^ isoacceptor that services rare AAA codons in Mtb (11). The depletion of this tRNA remodels the Mtb proteome in complex ways downstream of the initial codon-dependent effects (as evidenced by the many up- and downregulated proteins in Fig. 2). Our identification of targeted upregulation of two pathways, PDIM biosynthesis and ribosome biogenesis, among the numerous, complex circuits and processes in Mtb underscores the specificity of MazF-mt9 when remodeling Mtb physiology. Enhanced ribosome biogenesis is likely a consequence of ribosome stalling. MazF-mt9 acts in a manner distinct from the other well-characterized Mtb tRNase toxin VapC4 (17), undoubtedly because each has a unique tRNA target whose cleavage and depletion initiates distinct downstream reprogramming events. VapC4 cleaves tRNA^Cys^; its depletion mimics Cys starvation to activate the cysteine biosynthesis pathway. The pool of newly synthesized Cys feeds into production of mycothiol, the glutathione counterpart in Mtb that protects cells from oxidative damage (17). Therefore, toxin-mediated cleavage and depletion of a highly specific tRNA target directs the course of the toxin-specific phenotypic shift.

PDIMs are only present in the outer membrane of pathogenic mycobacteria and are essential for virulence (reviewed in reference 29), particularly during lung infection (52). Their influence on Mtb has been mostly noted during early infection, where they appear to modulate the innate immune response (53). Alveolar macrophages are the first immune cells to encounter Mtb (54). The Mtb infection cycle begins after inhalation of aerosol droplets into the deep lung. Instead of being a barrier to infection, alveolar macrophages patrolling the airway surface can serve as a reservoir for Mtb, which engages multiple strategies to adapt to, or alter, the usual bactericidal program after macrophage uptake of a pathogen. When bacterial pathogens are typically engulfed by the macrophage, the resulting phagosome undergoes gradual acidification, fuses with the lysosome, and is broken down by lysosomal enzymes. Mtb avoids killing upon macrophage uptake by blocking phagosome maturation by inhibiting both phagosome acidification (55–58) and lysosome-phagosome fusion (reviewed in reference 59). Mtb can also escape from the phagosome into the cytosol upon phagosome rupture (reviewed in references 60–62).

The precise role of PDIMs in this complex process of Mtb infection is still evolving. Consistent with a role for PDIMs in early infection, PDIM-deficient Mtb cells led to an attenuated infection in mice, in part due to increased sensitivity to reactive nitrogen intermediates in activated macrophages (63). Also, Cambier et al. used the closely related *M. marinum* to demonstrate that PDIMs are instrumental in evasion of immune clearing by alveolar macrophages because they mask the pathogen-associated molecular patterns (PAMPs) that decorate the bacterial cell surface (64, 65). Without PDIMs, TLR-responsive macrophages are recruited by PAMPs, and *M. marinum* is killed. The presence of PDIMs inhibits TLR signaling. Blocking this signaling pathway prevents recruitment of microbicidal macrophages and enables *M. marinum* survival in permissive macrophages recruited through a host chemokine receptor 2 (CCR2) pathway that is dependent on PGL acting as a ligand for macrophage receptors (64). However, H37Rv and its derivatives are PGL-deficient, complicating the ability to directly correlate studies on survival mechanisms in Mtb H37Rv with *M. marinum*. Nevertheless, several studies support a role for PDIMs alone, or in collaboration with PGL, in facilitating evasion of mycobacterial killing by macrophages (reviewed in reference 27).

The presence of PGL is associated with increased Mtb virulence in mice (66) and rabbit models (67). Although the strain we used does not have an intact *pks15/1* gene and cannot synthesize PGLs, we do see upregulation of many proteins and enzymes involved in its synthesis (Fig. 5C). MazF-mt9 is likely present in other strains that do carry a normal *pks15/1* gene, for example, a subset of the W-Beijing family; it is also present in *M. bovis* strains that make PGLs. Therefore, MazF-mt9 likely increases the synthesis of both PDIM and PGL in certain Mtb strains. Cambier et al. offered an interesting perspective on the trend toward the progressive dispensability of PGLs (64). Since tuberculosis is such an ancient disease, the presence of PGLs may have offered an infectivity advantage back when human populations were more dispersed. However, with densely populated urban centers commonplace throughout the modern world, this enhanced infectivity boost previously imparted by PGL may no longer be necessary.

Although Mtb inhibits both phagosome acidification and phagosome-lysosome fusion, PDIMs appear to only influence the former (55). No changes in phagosome-lysosome fusion were observed in a ∆*fadD26* deletion mutant lacking PDIMs (63), nor did PDIMs in wild-type or ∆*ppsE* PDIM-negative strains sort with a lysosomal marker (55). PDIMs are involved in phagosomal escape of Mtb to the cytoplasm, as well as the induction of both necrosis and autophagy in their macrophage host cells (68). PDIMs also contribute to Mtb impermeability to detergents (69), as well as acquired resistance to selected antibiotics (70–74).

In summary, our results place MazF-mt9 at the center of a coordinated metabolic response that is echoed in the transcriptome of bedaquiline-treated cells, thereby reinforcing the BONCAT-based evidence for suppression of lipid catabolism, activation of carbon-sparing pathways, and maintenance of central metabolism during host-imposed stress. Given the abundance of TA systems and the selective metabolic reprogramming triggered by MazF-mt9, our findings support a model in which diverse toxins act cooperatively to reshape Mtb physiology, enabling the bacterium to evade lethal immune pressures and sustain long-term persistence. Future studies of MazF-mt9 in macrophage- and animal-infection models will further define how these toxin-driven programs contribute to pathogen survival and immune modulation *in vivo*.

## MATERIALS AND METHODS

### Strains, plasmids, and reagents

All experiments were performed using Mtb mc^2^6206 (Δ*panCD* Δ*leuCD*), generously provided by William Jacobs Laboratory, Albert Einstein College of Medicine. Mtb cells were grown under constant shaking at 170 rpm at 37°C in 7H9 Middlebrook medium containing 1× OADC supplement (Millipore Sigma), 0.05% tyloxapol, and kanamycin at 25 μg/mL for plasmid selection. The media were supplemented to 50 µg/mL pantothenic acid and 100 µg/mL leucine. Construction of the pMC1s::*mazF-mt9* plasmid was previously described (11).

### Proteomics of newly synthesized proteins

To identify newly synthesized proteins by quantitative mass spectrometry, quadruplicate ±MazF-mt9 samples were induced for 9 days, and AHA (Anaspec Inc.) was added to the medium to a final concentration of 50 µM and incubated overnight. Fifty milliliter cultures were centrifuged at 2,000 *g* at 4°C for 5 min, and the cell pellets were washed with 1× PBS–0.05% tyloxapol two times to remove traces of the albumin-containing 7H9 medium. To extract the AHA-labeled proteins, cells were pelleted, resuspended in lysis buffer (2% CHAPS, 8 M urea), and lysed in a Precellys Evolution homogenizer. The lysates were pelleted at 12,000 *g* at 4°C for 10 min, and the AHA-labeled proteins contained in the supernatant were selectively captured using alkyne-coated agarose beads from the Click-iT Protein Enrichment Kit (Thermo Fisher), following the manufacturer’s protocol.

Tryptic digests were evaluated using a Thermo Q Exactive HF mass spectrometer and nanoflow LC system (Thermo Scientific), as described in Barth et al. (11). Data were analyzed with MaxQuant (version 2.6.2.0) with the Andromeda search engine. The type of LC-MS run was set to 1 (label-free). LC-MS data were searched against the NCBI *Mycobacterium tuberculosis* H37Rv reference proteome (accession AL123456.3) plus a common contaminant default database for MaxQuant. Protease was set as trypsin that allowed two missed cuts. Cysteine carbamidomethylation was set as a fixed modification, N-terminal acetylation and oxidation at methionine were set as variable modifications, and AHA incorporation in place of methionine was considered. A maximum of two variable modifications was allowed. The maxLFQ value was used for protein quantification (75). Protein and peptide false discovery rates (FDR) were set at 1%. The search results were further analyzed with Perseus (version 2.1.1.0). Reverse hits and common contaminants, as well as proteins identified only by modified sites, were filtered out. The maxLFQ was log_2_-transformed. Entries with at least three valid values in at least one sample group (induced or uninduced) and at least four valid values in all samples were kept. The missing values were imputed based on the total matrix using default settings (width = 0.3, shift = 1.8). The two-sample test, using the Student’s *t*-test (non-paired, two-tailed) and a permutation-corrected FDR of 5%, was used to calculate statistical significance between induced and uninduced samples.

### Lipid radiolabeling, extraction, and TLC

Four colonies of Mtb mc^2^6206 colonies carrying the pMC1s::*mazF-mt9* plasmid were individually resuspended in 100 μL of 7H9 Middlebrook medium, and equal parts (≈50 μL) of this bacterial suspension were inoculated in glass Erlenmeyer flasks (250 mL) containing 45 mL of 7H9 Middlebrook medium (10% OADC, 50 μg/mL pantothenic acid, 100 μg/mL L-leucine) with 25 µg/mL kanamycin. Then, 200 ng/mL of ATc (Takara Bio, USA) was added to half of the cultures (*n* = 3 or 4) to induce MazF-mt9 toxin expression (+ATc or induced group); ATc was replenished every 48 h to sustain toxin expression. Negative controls, in which ATc was not added, were also included (−ATc or uninduced group). Cultures were incubated at 37°C, under constant shaking at 170 rpm. Typically, the growth-arrest phenotype caused by MazF-mt9 toxin is apparent at day 3. Therefore, at 3, 4, 6, or 8 days following MazF-mt9 induction, we labeled 20 mL cultures in 50 mL conical tubes with 0.4 µCi/mL of [1,2-^14^C] acetic acid, sodium salt (Revvity), or propionic acid [1-^14^C] sodium salt (American Radiolabeled Chemicals, Inc.). Cells were harvested 48 h later by centrifugation (3,000 rpm, 10 min, room temperature), resulting in a total toxin induction time of 5, 6, 8, or 10 days at harvest. The cells were washed twice with ultra-pure water (10 mL), and the supernatant was discarded. Pellets were resuspended in 5 mL of chloroform:methanol (2:1, vol/vol) solution, and total lipids were extracted overnight at −20°C. The organic phase containing the lipids was transferred to a new tube, and the process was repeated with 2.5 mL of chloroform:methanol (2:1, vol/vol) solution for 2 h at 4°C. The two organic fractions were pooled, and 0.2 equivalents (of the total volume) of water were added to induce phase separation. The organic phase was carefully transferred to new tubes kept open in a fume hood until complete chloroform:methanol evaporation. The dry pellets were resuspended in 1 mL diethyl ether (Acros Organics) and incubated overnight at −20°C. ^14^C incorporation was then determined by scintillation counting. After that, the diethyl ether was evaporated in a fume hood, and lipids were dissolved in different volumes of chloroform:methanol (1:1, vol/vol) solution to achieve 1,000 counts per minute (CPM) per µL (CPM/µL) for samples in Fig. 6A through D or 10,000 CPM/µL in Fig. 6E and F. Ten thousand CPM (Fig. 6A through D) or 100,000 CPM (Fig. 6E and F) of ^14^C-labeled lipids (10 μL) were carefully spotted onto a 400 cm^2^ HPTLC silica gel sheet (Millipore Sigma). Finally, the lipids were resolved by TLC in a mobile phase of 9:1 hexane:diethyl ether (≈100 mL) (Fig. 6A through D) or 98:2 petroleum ether:ethyl acetate (Fig. 6E and F) and visualized with a Typhoon FLA 9500 laser scanner. The relative intensity of all PDIM bands was determined using the ImageJ 1.53k software. Then, the relative intensity of all PDIM bands in individual samples was combined and compared using a two-tailed, paired *t-*test or a Wilcoxon matched-pairs signed-rank test. The H37Rv pure PDIM control (bei RESOURCES catalog no. NR-20328) was resolved with 9:1 hexane:diethyl ether and visualized upon spray saturation with 10% phosphomolybdic acid hydrate in ethanol, followed by heating at 140°C for 10 min (76).

### Statistical analyses

Statistical analyses for growth profiles and volcano plots were performed using GraphPad Prism software 10.4.2.

## Data Availability

The mass spectrometry proteomics data set has been deposited to the ProteomeXchange Consortium via the PRIDE (77) partner repository with the data set identifier PXD052177.

## SUPPLEMENTAL MATERIAL

Figure S1 msystems.01814-25-s0001.docx Representative growth profiles of ±MazF-mt9 Mtb cells used for lipid labeling and PDIM TLCs.

Table S1 msystems.01814-25-s0002.xlsx MazF-mt9 BONCAT data.

Table S2 msystems.01814-25-s0003.xlsx MazF-mt9 DAVID output.

ASM does not own the copyrights to Supplemental Material that may be linked to, or accessed through, an article. The authors have granted ASM a non-exclusive, world-wide license to publish the Supplemental Material files. Please contact the corresponding author directly for reuse.
