# Supplementary material for: Proteomics reveals coordinated stress adaptation by a MazF toxin to conserve carbon, sustain central metabolism, and preserve PDIM biosynthesis in Mycobacterium tuberculosis
Source: mSystems. 2026 Mar 23;11(4):e01814-25. doi: 10.1128/msystems.01814-25 (PMC13098245; doi:10.1128/msystems.01814-25)
Supplement: Figure S1 — Representative growth profiles of ±MazF-mt9 Mtb cells used for lipid labeling and PDIM TLCs. [file msystems.01814-25-s0001.docx]

**SUPPLEMENTAL INFORMATION**

**Supplemental Fig. 1. Representative growth profiles of ±MazF-mt9 Mtb cells used for lipid labeling and PDIM TLCs.** **A** Early-log phase cultures (OD_600_ 0.1-0.2) were radiolabeled with 0.4 µCi/ml [^14^C] sodium acetate on day 3 (black arrow) post toxin induction with ATc and cells were harvested on day 5 for lipid extraction. Error bars represent the standard deviation from the average of four biological replicates. Asterisks indicate statistical significance between uninduced (-MazF-mt9; gray) and induced (+MazF-mt9; red) groups in a two-tailed paired *t*-test comparison (**p* < 0.05; ****p* = 0.0009). **B-E** Individual growth profiles of the four replicates averaged in panel A. **F** TLC of ^14^C-labeled lipids from Mtb cultures with (+) or without (-) MazF-mt9 expression spotted at origin showing PDIMs A and B resolved in hexane/diethyl ether (9:1; v/v). Total lipids were extracted from four biological samples (n = 4) for ±MazF-mt9. This is the original **nonenhanced** phosphorimager image to clearly demonstrate that fewer lipids in the four +MazF-mt9 samples were spotted and resolved compared to the control samples as indicated by their lighter intensity origin. **Note that since MazF-mt9 expression arrests cell growth, high levels of ^14^C incorporation into lipids are not attainable. G** Enhanced exposure of panel F. However, the lower levels of lipid spotted at the origin in the +MazF-mt9 samples relative to the −MazF-mt9 control are no longer apparent because the exposure is beyond the linear range. **H** Two exposures of TLC separation of 100 µg of pure H37Rv PDIM (bei RESOURCES) run for the same distance and resolved with the same solvent mixture as in panel E and F but visualized with 10% phosphomolybdic acid hydrate in ethanol and heated for 10 minutes at 140°C ([75](#_ENREF_75)). The solvent migration front appears as a dark gray line extending across the entire panel (not visible in panels F, G). PDIM A is most abundant in this pure PDIM batch; the PDIM B precursor is the lowest band. The middle intermediate band is sometimes present upon [^14^C] labeling.
